# Marginal Leakage of Endodontic Temporary Restorative Materials around Access Cavities Prepared with Pre-Endodontic Composite Build-Up: An In Vitro Study

**DOI:** 10.3390/ma13071700

**Published:** 2020-04-05

**Authors:** Atsushi Kameyama, Aoi Saito, Akiko Haruyama, Tomoaki Komada, Setsuko Sugiyama, Toshiyuki Takahashi, Takashi Muramatsu

**Affiliations:** 1 Department of Operative Dentistry, Endodontology and Periodontology, School of Dentistry, Matsumoto Dental University, Shiojiri 399-0781, Japan; 2 Section of Dental Hygiene, Tokyo Dental College Suidobashi Hospital, Tokyo 100-0061, Japan; saitouaoi@tdc.ac.jp; 3 Department of Operative Dentistry, Cariology and Pulp Biology, Tokyo Dental College, Tokyo 101-0061, Japan; akiharu@tdc.ac.jp (A.H.); komadat@tdc.ac.jp (T.K.); tmuramat@tdc.ac.jp (T.M.); 4 Division of General Dentistry, Tokyo Dental College Chiba Dental Center, Chiba 261-8502, Japan; setsukos@tdc.ac.jp (S.S.); totakaha@tdc.ac.jp (T.T.)

**Keywords:** endodontic treatment, pre-endodontic composite build-up, marginal leakage, endodontic temporary sealing material, thermocycling

## Abstract

This study aimed to examine the marginal seal between various commercial temporary restorative materials and exposed dentin/built-up composite. Sixty bovine incisors were cut above the cemento-enamel junction, and half of the dentin was removed to form a step, which was built up using flowable resin composite. The root canals were irrigated, filled with calcium hydroxide, and sealed using one of six temporary sealing materials (hydraulic temporary restorative material, temporary stopping material, zinc oxide eugenol cement, glass-ionomer cement, auto-cured resin-based temporary restorative material, and light-cured resin-based temporary restorative material) (*n* = 10 for each material). The samples were thermocycled 500 times and immersed in an aqueous solution of methylene blue. After 2 days, they were cut along the long axis of the tooth and the depth of dye penetration was measured at the dentin side and the built-up composite side. For the margins of the pre-endodontic resin composite build-up, the two resin-based temporary restorative materials showed excellent sealing. Hydraulic temporary restorative material had a moderate sealing effect, but the sealing effect of both zinc oxide eugenol cement and glass-ionomer cement was poorer.

## 1. Introduction

Since root canal treatment often requires multiple visits, it is necessary to maintain the condition of the tooth at the end of each treatment until the next visit. Additionally, temporary sealing of the access cavity plays an important role in maintaining the bactericidal action of the intracanal medicament [1].

Temporary restorative materials should render the seal of the access cavity wall moisture-proof to prevent marginal leakage. They are also required to withstand masticatory stress, have excellent esthetic properties, be chemically and physically stable, and be easy to manipulate [2]. Therefore, hydraulic temporary restorative material, temporary stopping material, zinc oxide eugenol cement, glass-ionomer cement and resin-based temporary restorative material are currently used singly or in combination for temporary sealing [3].

Many studies have been conducted on the marginal sealing effect of temporary endodontic sealing materials [3,4,5,6,7,8,9,10,11]. Most have compared the sealing ability of these materials under conditions in which the enamel remains within the four-step scoring criteria as described by Lee et al. [12]: (1) no visible dye penetration at the tooth/temporary filling interface, (2) dye penetration limited to the dentin–enamel junction, (3) dye penetration of up to half of the pulp chamber, and (4) dye penetration of over half of the pulp chamber. However, in actual clinical practice, treatment is often performed on teeth that have major loss of the dental crown structure, and in such cases, the cavity margin of the temporary restoration is often dentin. Additionally, in almost all cases of endodontic retreatment, there is no remaining dentin on the level of the gingival margin, because in these cases, the crown is often completely restored. To achieve absolute isolation with a rubber dam, pre-endodontic resin composite build-up is often performed [13,14]. The margin of the temporary restoration is between the temporary restorative material and the built-up resin composite. Only a few studies have examined the ability of temporary restorations to achieve sealing when dentin is exposed on the cavity surface [3,15]. Furthermore, no study has examined the effectiveness of temporary restorations in teeth with a resin composite build-up.

The purpose of this study was therefore to examine the ability to achieve marginal sealing between the various types of commercial temporary restorative materials and exposed dentin/built-up resin composite, simulating cases in which there is severe loss of the tooth substrate. The null hypotheses to be verified in this study are: (1) that there is no difference in the marginal sealing effect of the various temporary restorative materials, and (2) that the temporary sealing effect of the dentinal margin and the margin with the composite build-up is equal.

## 2. Materials and Methods

### 2.1. Pre-Endodontic Composite Build-Up

**Figure 1:**
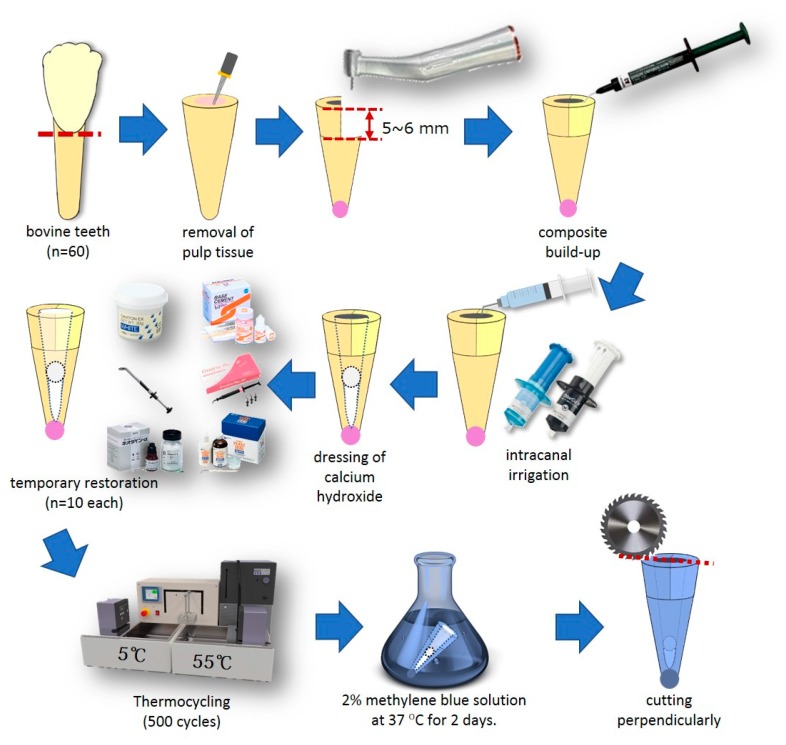
Schematic drawing of the study set-up.

The outline of this study is schematically shown in Figure 1. Sixty bovine mandibular incisor teeth (Yokohama Meat Corporation, Yokohama, Japan), which were frozen immediately after removal, were defrosted using running tap water immediately before use. After removing the periodontal tissue adhering to the bovine teeth, they were cut about 5 mm above the cemento-enamel junction, and the pulp tissue in the root canal was removed with a #80 K-file (Mani, Utsunomiya, Japan). Next, the mesial or distal half of the tooth stump was removed to prepare 5–6 mm steps using a diamond bur (Mani Dia-bur TF114, Mani, Utsunomiya, Japan) mounted on a high-speed micro motor handpiece (Ti-Max X 95, NSK, Kanuma, Japan). Self-etching primer and bonding resin (Clearfil Megabond 2, Kuraray Noritake Dental, Tokyo, Japan) was applied according to the manufacturers recommendations, light-cured with an LED light-curing unit (Demi Plus, Kerr, Orange, CA, USA), and a flowable resin composite (Tokuyama Estelite Universal Flow Quick Super Low A2, Tokuyama Dental, Kamisu, Japan) was built up to restore the missing portion of the tooth stump. After correction of the cavity wall shape using a diamond bur, the apex and its periphery were sealed with a self-curing acrylic resin (Unifast III, live pink, GC, Tokyo, Japan).

### 2.2. Intracanal Irrigation

For intracanal irrigation, 1 mL of a 3% NaClO solution (Chlorsid J, Ultradent Products, South Jordan, UT, USA) was placed in the root canal using a 1 mL disposable syringe (Terumo, Tokyo, Japan) attached to a disposable non-bevel needle (23 G × 1 1/4, Nipro, Osaka, Japan). After 3 min, the canal was washed with 2 mL of distilled water. Next, the root canal was filled with an 18% EDTA solution (Ultradent EDTA 18%, Ultradent Products, Ultradent Products, South Jordan, UT, USA), which was contained in a disposable syringe for 1 min, and washed again with 2 mL of distilled water. After suction of the remaining water in the root canal, the canals were dried with #80 absorbent paper tips (JM paper point, J. Morita, Tokyo, Japan).

### 2.3. Temporary Restoration

Calcium hydroxide (FUJIFILM Wako Pure Chemical, Osaka, Japan) was mixed with distilled water and inserted into the root canal up to about 12 mm below the cavity margin, and a dry cotton ball was inserted at least 10 mm below the cavity margin using a root canal plugger (YDM, Tokyo, Japan) equipped with a rubber stop to secure the depth. These specimens were randomly assigned to six groups (10 specimens in each), and the temporary sealing procedure was performed as below.

- Hydraulic temporary restorative material (Caviton EX, GC, Tokyo, Japan): an appropriate amount of material was collected with a dental plastic filling instrument, pressed into the access cavity, and hardened with a wet cotton ball.
- Temporary stopping material (Temporary Stopping, GC, Tokyo, Japan): a temporary stopping material was inserted into a stopping carrier (YDM, Tokyo, Japan). The carrier was heated with a gas burner, and the softened temporary stopping material was inserted into the access cavity and pressure-welded with a dental plastic filling instrument.
- Zinc oxide eugenol cement (Neodyne-α, Neo Dental Chemical Products, Tokyo, Japan): powder and liquid was mixed using a mixing pad and a stainless steel spatula under the recommended powder-to-liquid ratio, and filled into the access cavity using a C-R syringe Mark II (Centrix, Shelton, CT, USA). A polyester matrix strip (GC, Tokyo, Japan) was placed on the top and finger pressure was applied.
- Glass-ionomer cement (Shofu Base Cement [White], Shofu, Kyoto, Japan): powder and liquid was mixed using a mixing pad and a stainless plastic spatula under the recommended powder-to-liquid ratio, and filled into the access cavity using a C-R syringe. A polyester matrix strip was placed on top and finger pressure was applied. After initial curing, protective varnish (GC Fuji Varnish, GC, Tokyo, Japan) was applied to the surface of the applied cement.
- Light-cured resin-based temporary filling material (Evadyne Plus, Neo Dental Chemical Products, Tokyo, Japan): a dedicated tip was attached to a syringe, and the material was inserted directly into the access cavity. After a polyester matrix strip was placed, finger pressure was applied to the filled material, and the coronal surface was light-cured using an LED light-curing unit (Demi Plus, Kerr, Orange, CA, USA). Light-curing was applied for 20 s from each of the two lateral areas for a total of 60 s.
- Self-cured resin-based temporary filling material (Nishika Plast Seal Quick, Japan Dental Pharmaceutical Manufacturing, Shimonoseki, Japan): a mixture of powder and liquid was placed in the access cavity using a brush-on-technique. A polyester matrix strip was applied with finger pressure until completion of the hardening.

### 2.4. Thermocycling and Dye Penetration

After storing for more than 2 h at room temperature, the samples were stored in water at 37 °C for 48 h. The coronal cut surface was trimmed with #150 SiC paper to expose the cavity margin. Thereafter, the specimens were thermocycled for 500 cycles in water at 5 ± 1.5 °C and 55 ± 1.5 °C, with a dwell time of 30 s in each water bath. The thermocycled specimens were immersed in 37 °C water again for 72 h.

The samples were then air-dried, and the root surface and coronal enamel were covered with New Sticky Wax (GC, Tokyo, Japan). The area excluding the 1 mm around the cavity of the cut surface of the crown was covered with nail varnish. The samples were then kept still for 2 days in a 2% methylene blue aqueous solution at 37 °C.

After completion of the prescribed period, the samples were well rinsed with running tap water, and a portion 15–20 mm away from the cut surface of the crown was cut perpendicularly to the long axis of the tooth using a hard tissue cutting machine (KT 100, Maruto, Tokyo, Japan). Subsequently, they were cut along the long axis of the tooth.

### 2.5. Measurement of Linear Dye Penetration

The depth of dye penetration was measured in millimeters at 20× using a stereomicroscope (ZEISS Stemi 508, Zeiss, Oberkochen, Germany) for both the composite side and the dentin side. The section with the greatest depth of dye penetration was used as the final value for that specimen. If the methylene blue dye was penetrated beyond the bottom of the temporary restoration, the penetration depth was determined as 10 mm. The measurement of dye penetration was carried out by the same author (A.K.).

Data were analyzed using a two-way analysis of variance (ANOVA). If differences were found, pair-wise testing was performed using Tukey’s HSD test. The significance level was set to *p* < 0.05. Statistical analysis was performed using IBM SPSS ver. 18 statistical software (SPSS, Chicago, IL, USA).

## 3. Results

**Figure 2:**
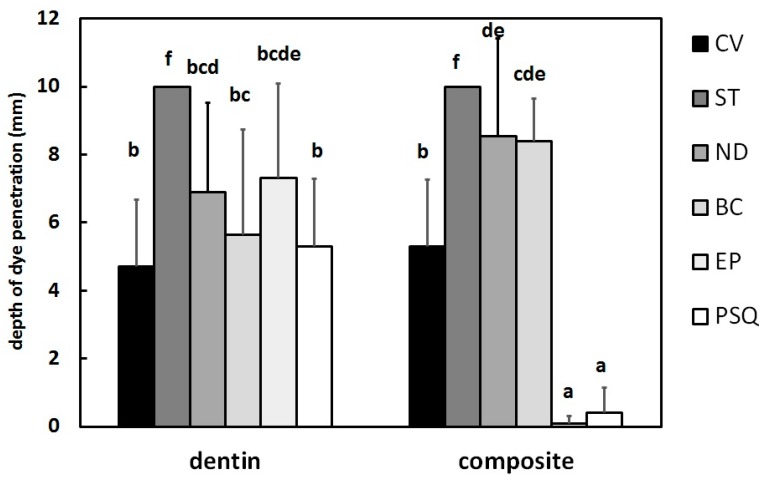
Depth of dye penetration in each group. Data are shown as the mean ± S.D. (*n* = 10); values, with the same letter indicating that there is no significant difference between the groups (*p* < 0.05). CV, Caviton EX (GC); ST, Temporary Stopping (GC); ND, Neodyne-α (Neo Dental Chemical Products); BC, Shofu Base Cement (Shofu); EP, Evadyne Plus (Neo Dental Chemical Products); PSQ, Nishika Plast Seal Quick (Japan Dental Pharmaceutical Manufacturing).

Figure 2 shows the depth of dye penetration in each group. According to the results of the two-way ANOVA, the type of temporary restorative material significantly affected the depth of dye penetration (*F* = 38.6, *p* < 0.001). Additionally, the type of cavity margin (composite *vs* dentin) also affected the penetration depth (*F* = 11.2, *p* = 0.001). Significant interaction was also found for the two factors (*F* = 20.8, *p* < 0.001).

### 3.1. Dentin Side

On the dentin side, no significant differences in the depth of dye penetration were found among Caviton EX (4.7 ± 2.0 mm; Range 2.5–8.0), Nishika Plast Seal Quick (5.3 ± 2.0 mm; Range 0.5–7.0), Shofu Base Cement (5.7 ± 3.1 mm; Range 1.5–10.0), Neodyne-α (6.9 ± 2.6 mm; Range 3.0–10.0), and Evadyne Plus (7.3 ± 2.8 mm; Range 2.0–10.0). However, Temporary Stopping penetrated the dye beyond the depth of the temporary restoration (< 10 mm) in all samples.

### 3.2. Composite Side

On the composite side, Evadyne Plus showed little dye penetration (0.1 ± 0.2 mm; Range 0.0–0.5) at the margin. There was no significant difference between Nishika Plast Seal Quick (0.4 ± 0.7 mm; Range 0.0–1.5) and Evadyne Plus (*p* > 0.05). Although Caviton EX (5.3 ± 2.0 mm; Range 3.0–10.0) had significantly greater penetration than Evadyne Plus and Nishika Plast Seal Quick, the depth was less than both Shofu Base Cement (8.4 ± 1.2 mm; Range 6.0–10.0) and Neodyne-α (8.6 ± 2.9 mm; Range 2.0–10.0). As on the dentin side, all specimens restored with Temporary Stopping showed penetration of dye beyond the depth of the temporary restoration.

### 3.3. Observation of Dye Penetration

**Figure 3:**
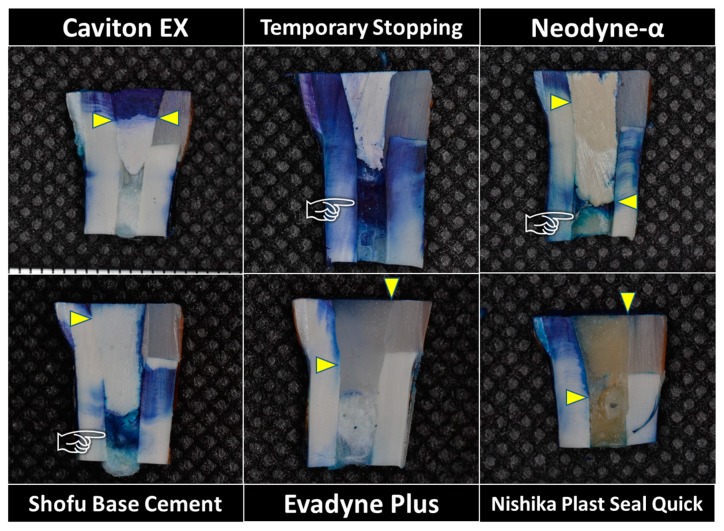
Representative photographs in each group after measurement of the penetration depth (yellow arrowhead). Note that the infiltration of dye on the cavity wall that was built up by resin composite could not be detected when a resin-based temporary sealing material was used, although deeper infiltration of the dye was observed on the cavity wall of the dentin side. It can be detected that a cotton ball placed directly below the temporary restoration was also stained by methylene blue in Temporary Stopping, Neodyne-α and Shofu Base Cement (pointer).

Representative photos of each group are shown in Figure 3. When Caviton EX was used for temporary sealing, the dye was absorbed into the Caviton EX to a depth of about 4–5 mm in each specimen. However, no further penetration was observed, and no staining of the cotton ball placed directly below the temporary restoration was observed. Temporary sealing with Neodyne-α and Shofu Base Cement showed deep dye penetration, especially on the cavity wall that was built up by resin composite. Although deeper infiltration of the dye was observed on the cavity wall of the dentin side when the temporary restoration was performed using resin-based material, it was almost undetectable on the cavity wall built up by composite.

## 4. Discussion

Prevention of contamination of the root canal by saliva, blood and microorganisms is important, not only during endodontic treatment, but also between each appointment before final obturation. To prevent contamination during endodontic treatment, rubber dam isolation is recommended. Rubber dams also prevent aspiration of instruments by the patient, retract/protect the soft tissue, and increase access to the working area [16]. However, rubber dam placement can be difficult when there is severe loss of tooth substrate, because the rubber dam clamp cannot be attached firmly to the tooth to be treated. Pre-endodontic composite build-up, which is a provisional restoration using an adhesive system and incremental building up of flowable resin composite, is recommended after the removal of carious tissue [13,14].

Temporary restorations are also necessary to prevent contamination between the end of each treatment and the next appointment. Although many studies have investigated the sealing ability of temporary restorative materials with an intact access cavity (i.e., with remaining enamel margins) [4,5,6,7,9,12], no studies have examined the sealing ability of temporary restorative materials in an access cavity interface of built-up composite. This in vitro study therefore evaluated the sealing ability of various types of commercial temporary restorative materials to access cavity interfaces of both dentin and built-up composite, to simulate cases of severe loss of the tooth substrate. ANOVA results led to rejection of the first null hypothesis, because the effect of marginal sealing varied according to the material used. The second null hypothesis was also rejected because the ANOVA results revealed differences in the sealing effect on the two types of cavity walls (composite build-up and dentin).

It is known that temperature changes in the oral cavity adversely affect the marginal seal of dental materials because the linear coefficient of thermal expansion of dentin is different to that of the restorative materials [17]. In the absence of thermal stress, temporary restorative materials can possess good sealing ability, even when there is no chemical adhesion to the tooth structure. This phenomenon may only be due to the wettability of the cavity wall and the strong cohesion of the interface [18]. However, the sealing ability has been reported to deteriorate under thermal stress. This is due to differences in the linear coefficients of thermal expansion between the tooth structure and the temporary restorative materials. Therefore, thermocycling tests are necessary to evaluate the marginal sealing ability of temporary restorative materials. This study conducted thermocycling for 500 cycles between 5 °C and 55 °C with a dwell time of 30 s, as in several previous studies [4,6,19]. These variables have been reported to simulate the intraoral temperature variations of eating and drinking [20].

Temporary Stopping is thermoplastic, easy to handle and easy to remove. However, it is not adhesive to the dentin surface. Additionally, its sealing properties are weak, because the coefficient of thermal expansion is large. Therefore, it is not generally used as the sole temporary restoration during root canal treatment. Deeper dye penetration and staining of the cotton pellets indicated the poorer marginal sealing ability and the refraction of these behaviors [21].

In the Caviton EX hydraulic temporary restorative material, calcium sulfate (gypsum) in the ingredients reacts with the water in saliva to progress hardening. In this study, the dye extended into the Caviton EX itself to a depth of about 5 mm. However, the marginal seal was excellent for both the dentinal margin and the margin of the composite build-up, because the cotton pellet immediately below the temporary restoration did not show any pigmentation. This material is hygroscopic—it expands when it comes in contact with moisture [22]. Therefore, this expansion permits the material to adapt more tightly to the cavity walls, thus providing a good seal under different conditions, including thermocycling [4].

Similar to Caviton EX, zinc oxide eugenol cement is also a material that expands on immersion in water. This cement has been recommended by Grossman as a temporary restorative material that can provide an effective seal [23]. Some studies have also reported that the sealing properties of zinc oxide eugenol cement are superior to those of hydraulic temporary restorative material [10,24,25,26], citing the tight adaptation of zinc oxide eugenol cement at the cavity–cement interface. Conversely, other previous studies concluded that the sealing ability of zinc oxide eugenol cement is inferior to hydraulic temporary restorative material [4,5,6,7,8,11]. These differences can be attributed to the different methods used for storing specimens: the studies suggesting the superiority of zinc oxide eugenol cement involved only immersion of the samples in water, whereas the studies asserting the superiority of hydraulic temporary restorative material evaluated the dye penetration after thermocycling. Therefore, it can be speculated that zinc-oxide eugenol cement is affected by thermal stress [27]. In this study, no significant difference was found between these two materials on the dentin side, but deeper infiltration of the dye was observed for zinc oxide eugenol than for Caviton EX on the built-up composite side. Zinc oxide eugenol might therefore not be recommended as a temporary restoration for teeth that have undergone pre-endodontic composite build-up. 

Glass-ionomer cement is known to adhere chemically to both enamel and dentin, and has a similar coefficient of thermal expansion to enamel and dentin [28,29]. The release of fluoride ions contained in the powder suggests that demineralization of the tooth structure may be prevented. Additionally, the compressive strength of glass-ionomer cement is greater than that of zinc oxide eugenol cement or hydraulic temporary restorative material [28,29]. Therefore, it is often used for temporary sealing after root canal treatment. Although the sealing ability of glass-ionomer cement at the dentinal margin is almost equivalent to that of Caviton EX, the dye was observed to penetrate to the base of the temporary restoration in almost all specimens. These results indicate that glass-ionomer cement does not form a chemical bond with the polymerized resin composite. Therefore, glass-ionomer cement is also not recommended for temporary restorations of teeth that have undergone pre-endodontic composite build-up.

Nishika Plast Seal Quick and Evadyne Plus are both soft resin-based temporary restorative materials. Resin-based materials always exhibit some shrinkage during polymerization, and do not adhere to tooth material. According to the report of Tamura et al. [30], the average coefficients of thermal expansion of Nishika Plast Seal Quick and Evadyne Plus are 141.5 × 10^−6^/°C and 161.2 × 10^−6^/°C, respectively. These values are large compared with that of dentin (10.2–11.4 × 10^−6^/°C) [28]. Therefore, these materials are generally not recommended for temporary restoration of the access cavity in root canal treatment. This study also revealed inferior results for the sealing ability of these materials in the dentinal margin.

However, there was negligible dye penetration at the margins of teeth with pre-endodontic composite build-up for both Nishika Plast Seal Quick and Evadyne Plus. These results support the clinical usefulness of resin-based temporary restorative material for cases in which pre-endodontic composite build up has been conducted around the entire circumference of the tooth. Nishika Plast Seal Quick was applied with a brush-on technique. The monomer liquid was applied to the cavity wall immediately before the monomer–polymer mixture was applied, to improve the adaptation to the cavity wall. The methacrylic acid ester contained in the liquid may have slightly dissolved the composite cavity wall resulting in a strong bond. Furthermore, abietic acid included as a tackifier might also have contributed to improve the adhesion [18]. Likewise, with Evadyne Plus, urethane dimethacrylate may have contributed the strong adhesion to the built-up composite.

The application of these materials has raised some concerns. For Nishika Plast Seal Quick, the base of the temporary restoration was softer than that of the sealed surface. This phenomenon indicates that the material was insufficiently polymerized. This could occur if a large amount of monomer was applied to the base of the cavity to prevent the formation of air bubbles, thus unbalancing the monomer/polymer ratio. It could also be difficult to apply the mixture of powder/liquid by a brush-dip technique to the deep cavity. In contrast, the base of the Evadyne Plus temporary restoration was hard. However, according to the manufacturer’s manual, this material can only be cured to a depth of 4.5 mm with 20 s of light curing. Unpolymerized residual monomer in the root canal may influence the results of root canal treatment. Therefore, further study of techniques for the application of resin-based temporary restorative material for temporary sealing of the root canal access cavity is required.

A minimum depth of 10 mm for all restorations was used in this study to comply with the recommendation for a minimum thickness of 3.5–4.0 mm for temporary restorations to prevent microleakage [4,31,32]. Although the sealing ability of Caviton EX was clinically acceptable, dye absorption was found throughout the bulk of Caviton EX restoration at a depth of about 4–5 mm. This demonstrates the need to make the temporary restorative material as thick as possible. This can be facilitated by use of a pre-endodontic composite build-up.

Most previous studies have evaluated the sealing ability of endodontic temporary restorative materials using intact teeth with ideal access preparations. However, most teeth requiring endodontic treatment have already lost a major component of the coronal structure as a result of severe caries and/or tooth fracture. Moreover, methylene blue dye used in this study might not be represented the variety of molecules in a real in vivo situation, where simultaneously multiple enzymes and factors are present [33]. Therefore, further laboratory and clinically studies are both needed to simulate a variety of clinical situations.

## 5. Conclusions

Within the limitations of the present in vitro study, the following conclusions were reached:

1. For dentinal margins, hydraulic temporary restorative material (Caviton EX), zinc oxide eugenol cement (Neodyne-α), glass-ionomer cement (Shofu Base Cement), and resin-based temporary restorative materials (Nishika Plast Seal Quick and Evadyne Plus) had similarly effective sealing ability. However, the sealing effect of Temporary Stopping was significantly lower than the other tested materials.
2. For the margins of pre-endodontic resin composite build-up, two resin-based temporary restorative materials (Nishika Plast Seal Quick and Evadyne Plus) showed excellent sealing properties. Caviton EX had a moderate sealing effect to both types of cavity wall. The sealing effect of zinc oxide eugenol cement (Neodyne-α) and glass-ionomer cement (Shofu Base Cement) was lower than Caviton EX.
